# Diagnostics of Primary Immunodeficiencies through Next-Generation Sequencing

**DOI:** 10.3389/fimmu.2016.00466

**Published:** 2016-11-07

**Authors:** Vera Gallo, Laura Dotta, Giuliana Giardino, Emilia Cirillo, Vassilios Lougaris, Roberta D’Assante, Alberto Prandini, Rita Consolini, Emily G. Farrow, Isabelle Thiffault, Carol J. Saunders, Antonio Leonardi, Alessandro Plebani, Raffaele Badolato, Claudio Pignata

**Affiliations:** ^1^Department of Translational Medical Sciences, Federico II University, Naples, Italy; ^2^Department of Clinical and Experimental Medicine, “Angelo Nocivelli” Institute for Molecular Medicine, University of Brescia, Brescia, Italy; ^3^Department of Clinical and Experimental Medicine, University of Pisa, Pisa, Italy; ^4^Center for Pediatric Genomic Medicine, Children’s Mercy Hospital, Kansas City, MO, USA; ^5^Department of Molecular Medicine and Medical Biotechnology, Federico II University, Naples, Italy

**Keywords:** primary immunodeficiencies, genetic diagnosis, targeted next-generation sequencing, whole exome sequencing, genotype–phenotype correlation

## Abstract

**Background:**

Recently, a growing number of novel genetic defects underlying primary immunodeficiencies (PIDs) have been identified, increasing the number of PID up to more than 250 well-defined forms. Next-generation sequencing (NGS) technologies and proper filtering strategies greatly contributed to this rapid evolution, providing the possibility to rapidly and simultaneously analyze large numbers of genes or the whole exome.

**Objective:**

To evaluate the role of targeted NGS and whole exome sequencing (WES) in the diagnosis of a case series, characterized by complex or atypical clinical features suggesting a PID, difficult to diagnose using the current diagnostic procedures.

**Methods:**

We retrospectively analyzed genetic variants identified through targeted NGS or WES in 45 patients with complex PID of unknown etiology.

**Results:**

Forty-seven variants were identified using targeted NGS, while 5 were identified using WES. Newly identified genetic variants were classified into four groups: (I) variations associated with a well-defined PID, (II) variations associated with atypical features of a well-defined PID, (III) functionally relevant variations potentially involved in the immunological features, and (IV) non-diagnostic genotype, in whom the link with phenotype is missing. We reached a conclusive genetic diagnosis in 7/45 patients (~16%). Among them, four patients presented with a typical well-defined PID. In the remaining three cases, mutations were associated with unexpected clinical features, expanding the phenotypic spectrum of typical PIDs. In addition, we identified 31 variants in 10 patients with complex phenotype, individually not causative *per se* of the disorder.

**Conclusion:**

NGS technologies represent a cost-effective and rapid first-line genetic approach for the evaluation of complex PIDs. WES, despite a moderate higher cost compared to targeted, is emerging as a valuable tool to reach in a timely manner, a PID diagnosis with a considerable potential to draw genotype–phenotype correlation. Nevertheless, a large fraction of patients still remains without a conclusive diagnosis. In these patients, the sum of non-diagnostic variants might be proven informative in future studies with larger cohorts of patients.

## Introduction

Primary immunodeficiencies (PIDs) represent a heterogeneous group of monogenic disorders including more than 250 genetically defined diseases, mostly identified in recent years (1–3). A significant contribution to this rapid evolution is due to the integration of functional studies with the use of next-generation sequencing (NGS) technologies. The specific objective of this strategy is the identification of putative pathogenic alterations in known or novel genes implicated in well-defined biological pathways (4–7). However, despite the breadth of current knowledge, the diagnostic approach used for the identification of genetic causes underlying PID appears inefficient and time-consuming as the majority of PID cases remain without a diagnosis even after extensive clinical and genetic investigations (8). Moreover, even in a single PID gene mutation, a genotype–phenotype correlation is often missing, because various clinical phenotypes may be related to the same genetic defect and *vice versa* (9, 10). Nevertheless, early diagnosis in severe forms of PIDs is crucial to establish a proper treatment and improve the overall outcome (11, 12). In the traditional approach to PIDs, the molecular diagnosis has long been based on the sequencing of multiple genes by the Sanger method, a time-consuming strategy, which often results in delayed diagnosis (13).

Next-generation sequencing technology has become a valuable first-line diagnostic tool for the timely diagnosis of genetic disorders, in particular for complex clinical presentation (14). NGS allows rapid, cost-efficient, accurate, and high-throughput sequencing of millions of DNA fragments in a reasonably short time. Whole exome sequencing (WES) is limited to the coding regions and splice junctions of the genome, which despite accounting for only ~2% of the genome, contain about 85% of genetic alterations known as responsible for human diseases (15).

In PIDs, a targeted sequencing approach, restricted only to specific genes or to specific regions of interest is a reasonable possibility to identify putative pathogenic variants that explain a specific disorder, their related altered biological pathway, and the array of genes that are transcribed. This can be the first-line alternative as it involves a smaller dataset than WES or whole genome sequencing (WGS) and is easier for the management of the datasets (16–18). However, as the spectrum of distinct clinical entities and the presenting phenotypes are expanding because of the discovery of novel genes and of the identification of wider clinical phenotypes, the differential diagnosis and subsequent diagnostic targets must improve in parallel. This perspective makes the whole exome or genome sequencing strategy attractive options for the diagnosis of patients with PID (19).

Here, we report on a case series of 45 complex or atypical clinical phenotypes that were suggestive of PID, but difficult to diagnose in a timely manner using the current diagnostic procedures. Our aim was to use current NGS technology to help diagnose PID in this cohort of patients. We used T-NGS and WES in 27 and 18 patients, respectively.

## Materials and Methods

### Patients

We have studied 45 patients with a clinical history highly suggestive of a primary immunological defect, that were heterogeneous for ethnic origin, age, and sex. The patients were selected on the basis of clinical features and abnormal immune parameters (20). The clinical criteria included one or more of the following features: opportunistic infections, granuloma, chronic mucocutaneous candidiasis (CMC), intractable diarrhea, bronchiectasis, and severe autoimmunity. These symptoms were associated, in some patients, with non-immunological features. In six patients, a positive family history for a similar phenotype was observed. Clinical criteria were considered if associated with one or more of the following quantitative and/or qualitative immunological abnormalities: abnormal lymphocyte subsets (absolute count <2 SD of normal values according to ESID criteria); proliferative response to mitogens <10% of the levels measured in the control subject; absent/poor specific antibody response; hypogammaglobulinemia; elevated IgE levels (>2000 kU/l); severe impairment of cytolytic activity; and alteration of class switch recombination (CSR) with or without hyper-IgM. PID patients, selected on the basis of the above specified criteria, were subsequently grouped on the basis of NGS results, functional alterations, and consistency between genotype, phenotype, and immune assays.

The study was approved by the Institutional Ethical Committee “Carlo Romano” of Federico II University and by Ethical Committee of Spedali civili (Brescia) and conducted after informed consent was obtained.

### DNA Extraction and Sequence Capture Array Design

Genomic DNA was isolated from peripheral blood lymphocytes with QIAamp DNA Blood Mini Kit (Qiagen, Hilden, Germany). Quantity and quality were determined through the Epoch Microplate Spectrophotometer (BioTech Instruments, Winooski, VT, USA). A panel of 571 genes, including 68 genes known or predicted to be related to PIDs and/or immune regulation, was sequenced (Table S1 in Supplementary Material). Basically, broad searches in literature, PubMed queries and expert suggestions defined the gene panel. We used BioMart (Ontario Institute for Cancer Research/European Bioinformatics Institute) to retrieve the coordinates of all exons for the specified genes from Ensembl. Coordinates were based on the current human reference genome (hGRC37, hg19).

### Next-Generation Sequencing and Bioinformatics Analysis

Samples were sequenced using either a targeted panel of 571 (TaGSCAN v2.0) or WES for enrichment. Briefly, TaGSCAN samples were prepared for sequencing using Illumina’s TruSight Inherited Disease panel according to manufacturer’s protocols (Illumina, San Diego, CA, USA). Samples were sequenced to at least 2.5 GB on an Illumina MiSeq with TruSeq MiSeq V3 reagents, yielding paired 250 nucleotide reads. Samples were prepared for exome sequencing using the TruSeq HT library preparation kit (Illumina; San Diego, CA, USA) followed by exome enrichment using the xGen Exome Research Panel V1.0 (Integrated DNA Technologies; Coralville, IA, USA) according to manufacturers’ protocols. Paired-end 2 bp × 125 bp sequencing was completed on an Illumina HiSeq 2500 instrument in high output mode using V4 Chemistry. Samples were sequenced to at least 7 GB of data resulting in a minimum target coverage of 50×. For all samples, sequence was assembled, aligned to reference gene sequences based on human genome build GRCh37/UCSC hg19, and analyzed using custom-developed software, RUNES and VIKING (21).

Alignment, variant calling, and analysis were performed, as described previously (21). In detail, variants were filtered by frequency and variant category (22). Variant analysis was confined to coding and splice variants with a minor allele frequency (MAF) of 1% or less in the CMH internal database, in the Exome Variant Server (EVS)^1^ and Exome Aggregation Consortium (ExAC).^2^ Other tools used to restrict NGS analysis to a set of gene-associated regions relevant to clinical presentations, was symptom- and sign-assisted genome analysis (SSAGA), which mapped the clinical features in ill neonates and children to disease genes, and Phenomizer databases (21, 23). Sorting Intolerant From Tolerant (SIFT) and Polymorphism Phenotyping v2 (PolyPhen2) have been used to provide information on the impact of the variants.

### Functional Assays and Sanger Validation

Functional assays were performed to prove the impact of variants identified by NGS. Functional studies included lymphocyte proliferation assays performed through [^3^H]thymidine incorporation assay, natural killer cell-mediated lysis of target cells and lymphocyte apoptosis induced by FAS death receptor cross-linking, evaluated through flow cytometry, cytokines production after toll-like receptors (TLR) stimulation, evaluated through real-time PCR. Furthermore, protein expression was evaluated through western blot analysis and standard procedures. After identification of genetic variants that were predicted to be damaging, along with consistent genotype–phenotype correlation, mutations were validated by Sanger sequencing using standard protocols. In 9 patients, we detected 11 variants that were confirmed by Sanger sequencing.

### Additional Phenotype-Based Variant Filtering Criteria

In order to identify potential causal mutations, annotated variants were further prioritized based on the following criteria:

– homozygous or heterozygous variants already reported that were related to any immunological clinical phenotype were selected to be further investigated by functional studies and validated by Sanger method;
– variants in genes implicated in a molecular pathway related to the phenotype were considered if associated with any functional alteration, which was even partially consistent, with the clinical phenotype;
– all the genetic variants of genes that were probably unrelated to the molecular pathway suspected to be involved in the pathogenesis of the disease were excluded from the functional studies, but reported in an *ad hoc* repository.

## Results

### NGS of 45 Patients

Genomic DNA from 27 patients was enriched for all exons from 571 genes, including genes involved in immunological pathways, while DNA from 18 patients was enriched for all nucleotides of the exome. DNA from six patients, analyzed with TaGSCAN, was subsequently sequenced for the entire exome and the bioinformatic analysis of data results is currently ongoing. For TaGSCAN, 98.9% of base pairs targeted had at least 10× coverage. Mean depth of coverage was 580×. WES covered 97.05% of exonic regions at 10× or greater on average. The overall analytic sensitivity for single nucleotide variants (SNVs) was 98.7%.

The results of NGS exon sequencing are shown in Tables 1–4. After bioinformatics analysis and filtering, the potential causative nucleotide changes needed to be further evaluated in each individual patient (Table 5). All of these variants were SNVs or small indel variants, with no other alteration such as large deletions or insertions identified.

**Table 1:** **Genetic variants associated with typical PID**.

Patient	NGS method	Gene	Mutation	Protein	Zygosity	Inheritance	Clinical and immunological phenotype
001	T-NGS	*CD40LG*	c.373C>T	p.His125Tyr	Hom	XL	Severe hypogammaglobulinemia with hyper-IgM, neutropenia, *P. jirovecii* pneumonia, CMV infection, intractable diarrhea
002	WES	*STAT1*	c.847T>A	p.Leu283Met	Het	AD	Chronic mucocutaneous candidiasis, recurrent pneumonia, hypothyroidism, lymphopenia, poor vaccine response
003	WES	*BTK*	c.1105C>T	p.Leu369Phe	Hom	XL	Agammaglobulinemia
004	T-NGS	*JAK3*	c.856C>T	p.Gln286Ter	Hom	AR	T^−^B^+^NK^−^SCID, chronic diarrhea, poor proliferative response to mitogens, IgA deficiency

*CD40LG and JAK3 variants were identified with T-NGS and STAT1 and BTK variants identified with WES. These variations were found in patients with clinical phenotype of classic well-defined PIDs*.

**Table 2:** **Genetic variants associated with novel features of PID**.

Patient	NGS method	Gene	Mutation	Protein	Prediction score		Zygosity	Inheritance	Clinical and immunological phenotype
					SIFT	PolyPhen2			
005	T-NGS	*MYD88*	c.192_194del	p.Glu66del	–	–	Hom	AR	Chronic yersiniosis and terminal ileitis, recurrent severe cutaneous granulomatous abscesses, hyper IgE, hypereosinophilia, neutropenia
006	WES	*PLDN*	c.232C>T	p.Q78X	–	–	Hom	AR	Partial oculocutaneous albinism, nystagmus, recurrent cutaneous infections, thrombocytopenia, leukopenia, NK deficiency
007	WES	*DOCK8/CLEC7A*	c.3193delA	p.Ser1065Ala	–	–	Hom/Hom	AR	Intractable diarrhea, eczema, malignancy, food allergies, hyper IgE, lymphopenia
				X17/p.Tyr238X					

*MYD88 variant was identified through T-NGS in a patient with atypical features of MyD88 deficiency, presenting with chronic yersiniosis. PLDN variant was identified through WES in a patient with incomplete features of Hermansky–Pudlak type II syndrome and impairment of NK cytolytic activity. DOCK8/CLEC7A variants were identified for the first time in a patient with intractable diarrhea, malignancy, and features of Hyper IgE syndrome*.

**Table 3:** **Genetic variants potentially involved in the immunological features**.

Patient	NGS method	Gene	Mutation	Protein	Prediction score		Zygosity	Major clinical features
					SIFT	PolyPhen2		
8	T-NGS	*UNC13D*	c.335G>C	p.Cys112Ser	0.38	0.832	Het	Acute lymphoblastic leukemia treated with allogeneic HSCT, ethmoiditis, recurrent lymphadenopathy, autoimmune cytopenia, arthritis, hypogammaglobulinemia, hyper-IgM, IgA deficiency
		*CASP10*	c.683C>T	p.Pro228Leu	0.07	0.071	Het	
9	T-NGS	*CASP10*	c.1202_1208del	p.Cys401LeufsTer15	–	–	Het	Alopecia universalis, hyperthyrotropinemia, type I diabetes mellitus, dental enamel hypoplasia, developmental delay, short stature, candidiasis, hepatomegaly, multiple skeletal abnormalities, myopia, dysmorphic features, microcephaly, abnormal FAS-induced apoptosis. Hyper IgE
10	T-NGS	*DOCK8*	c.1907A>G	p.Lys636Arg	0.4	0.057	Het	Inflammatory bowel disease, short stature, aspergillosis, EBV infection, low CD4^+^ lymphocyte subset, increased CD4 CD8 double-negative T cells, normal antibody response
		*TLR3*	c.2672A>G	p.His891Arg	0.01	0.309		
11	T-NGS	*ADA*	c.377C>A	p.Pro126Gln	0	0.999	Het	T^−^B^+^NK^−^ SCID treated with bone marrow transplantation, *P. jirovecii* pneumonia, recurrent otitis, absent ossicular bone with hypoacusia of the right ear, mild brain, and cerebellar atrophy, speech delay, scoliosis
			c.1047A>G	p.K349=	–	–		
		*ERCC6*	c.3262A>G	p.Ser1088Gly	0.48	0.001		
			c.2697G>A	p.T899=	–	–		
12	T-NGS	*AP3B1*	c.787G>T	p.Gly263Cys	0.05	0.932	Het	Interstitial lung disease CMV infection, esophageal candidiasis, strabismus, abnormal expression of perforin in NK cells, reduction of CD4^+^ cells with increase of CD19^+^, normal proliferative response to mitogens, normal antibody response
		*PRF1*	c.695G>A	p.Arg232His	0.02	0.991		
			c.272C>T	p.Ala91Val	0.01	0.808		
		*ADAMTS13*	c.2701G>T	p.Ala901Ser	0.61	0.049		

*In this group of patients, T-NGS allowed to identify multiple variants not causative of a specific PID, but with a possible impact on the disease*.

**Table 4:** **Genetic variants not causative of PID with undetermined impact on the disease**.

Patient	NGS method	Gene	Mutation	Protein	Prediction score		Zygosity	Major clinical features
					SIFT	PolyPhen2		
13	T-NGS	*CFTR*	c.2991G>C	p.Leu997Phe	0.08	0.18	Het	Late onset hypogammaglobulinemia, recurrent pneumonia, bronchiectasis, chronic sinusitis, cervical and mediastinal lymphadenopathy, recurrent abdominal pain, hepatomegaly with low grade steatosis, splenomegaly
14	T-NGS	*SYCE2*	c.577G>A	p.Val193Met	0.28	0	Het	t(11;18)MLT1-AP12 gastric maltoma HP^+^, persistent oral candidiasis, sinusitis; lung cystis, chronic cough, recurrent fever, hypereosinophilia, recurrent itch, recurrent myofasciitis, hyper-IgM, altered somatic hypermutation, absent CD19^+^CD20^−^ IgG^+^ (mature), low CD19^+^CD27^+^IgM^+^ (memory), absent CD19^+^CD27^+^IgM^−^ (switched memory)
		*LYST*	c.10235G>A	p.Arg3412His	0.02	0.997		
15	T-NGS	*ATR*	c.5257A>G	p.Ile1753Val	0.16	0.403	Het	Severe aplastic anemia, hepatomegaly, *Legionella* sp. and *Aspergillus* recurrent pneumonia, metacarpal deforming alterations with bone demineralization, abnormal lymphocyte proliferation, dilated cardiomyopathy, early retinopathy
		*ARSA*	c.869G>A	p.Arg290His	0.55	0.959	Het	
		*CASP10*	c.683C>T	p.Pro228Leu	0.07	0.071	Het	
		*IKBIKG*	c.1165C>T	p.Pro389Ser	0.29	0.041	Het	
		*MEFV*	c.460T>C	p.Ser154Pro	0.04	0.001	Het	
		*SP110*	c.1114C>T	p.Arg372Ter	–	–	Het	
		*UNC13D*	c.335G>C	p.Cys112Ser	0.38	0.852	Het	
16	T-NGS	*ATRX*	c.2247_2249del	p.Ser750del	–	–	Het	Autoimmune adrenal insufficiency, autoimmune thyroiditis, lymphadenopathy, autoimmune thrombocytopenia, and neutropenia
			c.2133_2135del	p.Ser712del	–	–	Hom	
		*MYD88*	c.10_28del	p.Ala6ProfsTer39	–	–	Het	
		*DOCK8*	c.2920C>A	p.His974Asn	0.34	0.001	Het	
			c.3016C>A	p.His1006Asn	0.33	0.003	Het	
			c.3220C>A	p.His1074Asn	0.33	0.003	Het	
17	T-NGS	*TYK2*	c.3488A>G	p.Glu1163Gly	0.27	0.006	Het	Hypogammaglobulinemia, familial IgA deficiency, hyper IgE, multiple bronchiectasis, candidiasis
18	T-NGS	*TLR3*	c.634-10C>A	–	–	–	Het	Familial IgA deficiency, multiple bronchiectasis, recurrent respiratory infections, low IgM levels
			Intron					
19	T-NGS	*CASP10*	c.1094A>C	p.Tyr365Ser	0	0.982	Het	Mild hypogammaglobulinemia, undetectable CD16^+^ lymphocyte levels, pervasive developmental disorder
		*ERCC5*	c.2375C>T	p.Ala792Val	0	1	Het	
		*GJC2*	c.256G>A	p.Val86Ile	0.13	0.825	Het	
		*PEX26*	c.728C>T	p.Ala243Val	–	–	Het	
		*RAB23*	c.536A>C	p.Glu 179Ala	–	–	Het	
		*UBR1*	c.3290C>T	p.Thr1097Met	–	–	Het	
20	T-NGS	*CD3-ZETA*	c.301C>T	p.Gln101Ter	–	–	Het	Hypogammaglobulinemia, recurrent pneumonia, transient alopecia, behavioral disorders, oropharyngeal candidiasis
		*OCRL*	c.2032A>G	p.Ser678Gly	0	0.986	Hem	
21	T-NGS	*PKHD*	c.5125C>T	p.Leu1709Phe	0.15	1	Het	Multi-organ failure and hypocalcemia during EBV infection, persistent EBV infection, kidney single cystic formation, hyper IgE, normal antibody response and proliferative response to mitogens, normal perforin intracytoplasmic expression, normal degranulation assay, reduced production of IFNγ
		*NPHP3*	c.2864del	p.Asp955ValfsTer2	–	–	Het	
		*G6PC*	c.634A>G	p.Ile212Val	0.88	0.01	Het	
					

22	T-NGS	*RAB27*	c.418C>G	p.Gln140Glu	1	0.24	Het	Recurrent fever, oral aphthous, diarrhea, laterocervical
		*LYST*	c.6482A>C	p.Glu2161Ala	0.03	0.002	Het	lymphadenopathy, hepatomegaly, increased level of amyloid protein, recurrent pneumonia, increased level of IgA, hyper IgE increased double-negative lymphocytes, normal functional Fas assay

*In this fourth group, T-NGS led to identify multiple genotypic alterations in patients showing complex phenotypes, which could not fit into defined clinical syndromes. In this group, no genotype–phenotype relationship was possible. However, since these cases are extremely rare, these data should likewise be collected in a database for future studies pointing to the sum of alterations on the whole*.

**Table 5:** **Gene variants confirmed by Sanger sequencing, functional assays and/or previous report**.

Gene	Mutation	Sanger confirmation	Functional assays	Reference
*CD40LG*	c.373C>T	Yes	Absent expression of CD40L after stimulation	(24)
*BTK*	c.847T>A	Yes	NA	(25)
*STAT1*	c.1105C>T	Yes	IFNα- and IFNγ-induced increased level of pSTAT1	(26)
*JAK3*	c.856C>T	Yes	Abnormal proliferative response to mitogens	–
*MYD88*	c.192_194del	Yes	Reduced levels of IL-6, IL-1, CCL2, and CCL3 after TLR stimulation with LPS, IL-1, TNFα; rescue of IL-1β and LPS responsiveness after WT *MYD88* gene transfection	(27, 28)
*PLDN*	c.232C>T	Yes	Absent PLDN protein expression	(29, 30)
*DOCK8/CLEC7A*	c.3193delA	Yes	Low level of DOCK8 protein expression	(31)
*UNC13D*	c.335G>C	NA	NA	(32)
*CASP10*	c.683C>T	NA	NA	–
*CASP10*	c.1202_1208del	Yes	Abnormal Fas-induced apoptosis in PHA-activated T cells	–
*DOCK8*	c.1907A>G	NA	–	–
*TLR3*	c.2672A>G	NA	Normal INFγ production after TLR3 stimulation	–
*ADA*	c.377C>A	NA	Abnormal proliferative response to mitogens	(33, 34)
*ERCC6*	c.3262A>G	NA	NA	–
*AP3B1*	c.787G>T	NA	NA	–
*PRF1*	c.695G>A	Yes	Reduced expression of perforin in NK cells	(35)
*ADAMTS13*	c.2701G>T	NA	NA	–

*For each variant, the Sanger confirmation, the functional assays, and references for variants already published were reported*.

*NA, not applied*.

A satisfying molecular diagnosis of PID was achieved in 7 of the 45 patients (16%), including 3 patients with an atypical presentation. Three causative variations were identified through T-NGS, while five through WES. On the basis of the genetic findings and of the clinical phenotypes, patients were divided in four groups. Group 1 included subjects with a diagnostic genotype that was previously associated with an immunological and/or clinical phenotype already reported for that genetic disease. Group 2 included subjects with a diagnostic genotype associated with novel or atypical clinical features of a genetic disease. In the group 3, NGS revealed multiple genetic variants that were consistent only with some features of the clinical and immunological phenotype. Group 4 included patients with a complex unclassified disorder that was associated with multiple genetic variants, each of them, individually, was not *per se* causative of the disorder. However, regarding the variants of groups 3 and 4, it should be pointed out that, since parental cosegregation studies were not performed, the variants may not be truly disease-causing or disease-modifying alterations, especially if inherited together from an unaffected parent.

The first group included four subjects who have been identified for a genetic defect associated with a known well-established immunological and/or clinical phenotype of PID (Table 1). In detail, variants in *CD40LG, BTK, STAT1* genes were already reported in literature as pathogenic (24–26). The *JAK3* nonsense variant was predicted to result in nonsense-mediated decay resulting in no protein expression. This mutation, though not previously reported, was fully congruent with the classic JAK3–SCID phenotype observed in multiple affected family members who shared the same genotype (proband and two siblings).

The second group included other three subjects in whom genetic variants were found as associated with novel phenotypic features, in previously characterized phenotypes (Table 2). Specifically in this group, patient 005 was found to carry a homozygous mutation in *MYD88* (c.192_194del; p.Glu66del), previously reported in several MYD88 deficiency affected families (27, 28). This patient was of a Rom ethnicity and had inherited the deletion from his unaffected consanguineous parents. But, early infant deaths due to severe infections were observed in the same family pedigree. Functionally, known mutations result in impairment of cytokine production after TLR stimulation (36, 37). Patients 006 and 007 were found to carry homozygous deleterious mutations in *PLDN* and *DOCK8/CLEC7A* gene, respectively, whose phenotypic peculiarity has been described in detail (29, 31).

The third group of subjects included five patients who carried multiple heterozygous variants affecting genes expressed in the hematopoietic system that were not consistent with a specific PID (Table 3). However, some of these patients might harbor a second mutation that was not identified by NGS or Sanger sequencing. In patient 008, we observed a heterozygous, probably, pathogenic variant in *UNC13D* (c.335G>C p.Cys112Ser), and a heterozygous, most likely benign variant in *CASP10* gene (c.683C>T p.Pro228Leu; exon 10 not covered). This patient had a clinical phenotype consistent with an autosomal dominant type II ALPS (ALPS-II), associated with hypogammaglobulinemia and acute lymphoblastic leukemia. Even though the *CASP10* variant alone in the *in silico* prediction programs, PolyPhen2 and SIFT, was predicted to be tolerated and was observed in 35 healthy individuals (ExAC), its significance in association with the second deleterious variant in *UNC13D* has never been described and could be potentially relevant. In fact, this UNC13D variant has been previously reported in association with heterozygous mutation of *FAS* and is considered a disease modifier for ALPS (32). In patient 009, we found by T-NGS a heterozygous frameshift variant in *CASP10* causative of ALPS-II and categorized as likely pathogenic according to American College of Medical Genetics (ACMG) criteria (22). His immunological phenotype was characterized by very high IgE levels (>2000 IU). The functional analysis of Fas-induced apoptosis in PHA-activated T cells from the patient confirmed that the apoptotic pathway was impaired, since cell apoptosis upon triggering of Fas was impaired (92% survival; normal values: median 60%, 95th percentile 82%). His brother, who presented with similar manifestations, died early in life because of hemophagocytic lymphohistiocytosis. In addition, the patient 009 also had additional clinical features, such as developmental delay, microcephaly, peculiar facial dysmorphism, and skeletal abnormalities, which could not be at moment demonstrated or excluded to be directly explained by the variant. In patient 010, we found two heterozygous variants of unknown significance in *DOCK8* and *TLR3* with good coverage of each gene and no second variant. The *TLR3* variant had multiple lines of computational evidence supporting a deleterious effect on gene/protein. Further studies are ongoing to find a possible correlation between the association of the two variants with the clinical phenotype, which was characterized by inflammatory bowel disease, susceptibility to viral infections, aspergillosis, T-cell lymphopenia, and increased CD4 and CD8 double-negative T cells. Alternatively, a second unidentified mutation of DOCK8 might account for the clinical manifestations of the patient. In patient 011 with a T^−^B^+^NK^−^ SCID phenotype associated with deafness, microcephaly, brain and cerebellar atrophy, developmental delay, NGS revealed 2 heterozygous mutations of *ADA* gene, located in the same allele, the first one being likely pathogenic, and a further heterozygous variant of unknown significance of *ERCC6* gene. The latter gene, which encodes for Cockayne syndrome B protein, has been recently described as essential for postnatal neuronal differentiation and neuritogenesis (38). Intriguingly, the patient showed also some neurological manifestations, such as macrocephaly and moderate grade cerebral atrophy. In patient 012 affected with hemophagocytic lymphohistiocytosis and marked reduction of perforin expression in NK cells, we identified two heterozygous mutations in PRF1 that were located in the same allele as shown by Sanger sequencing. In the same patient, we have also detected heterozygous variants in AP3B1 and ADAMTS13 genes that have been implicated in NK activity as well.

The last group of patients included 10 patients with a complex disorder that was not typical of any known syndrome and was associated with multiple genetic variants, each of them was not *per se* causative individually of that disorder (Table 4). In this group, it was not possible to draw any correlation between the genetic variations that were detected and the pathogenesis of the disorders. However, these alterations of unknown biologic significance were found in several genes implicated in immunological functions at different extent.

In the remaining 23 patients who were analyzed by NGS, including 9 studied by T-NGS and 15 by WES, we could not identify any candidate variants. These unsolved datasets will be re-analyzed when new bioinformatics tools become available and as new disease genes are described.

## Discussion

The advent of NGS technologies has given the possibility to physicians to investigate multiple genes assay, to provide great opportunities for diagnosing patients affected with complex disorders of the immune system, and to increase our knowledge on the pathogenesis of these genetic disorders (39).

**Figure 1:**
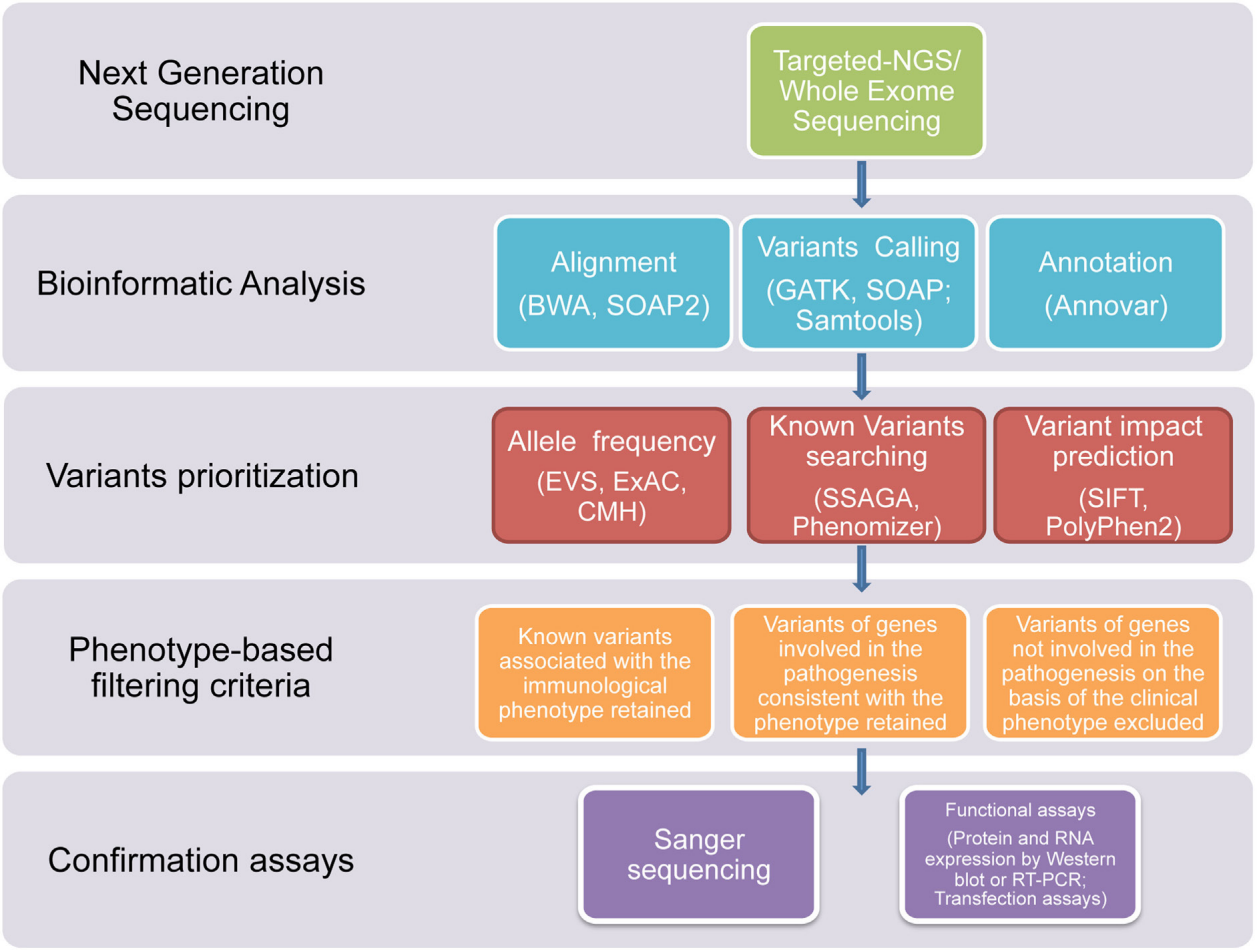
**Flowchart of the filtering strategy**. A schematic overview of the approach/clear-cut strategy used to filter variants identified through T-NGS or WES in order to identify potentially causative mutations.

In this study, we used NGS technologies to identify potential disease-causing mutations in patients affected with clinical phenotypes highly suggestive of a PID, which were still not diagnosed after using traditional sequential Sanger sequencing procedures. Thanks to this novel diagnostic approach, we report that a definitive diagnosis of PID was achieved in a timely manner in 7 out of the 45 subjects. In three patients, the diagnosis was achieved through T-NGS, while, in the other four patients, the diagnosis was reached by WES. In all these subjects, the application of a clear-cut filtering strategy, consisting of targeted sequencing, bioinformatics analysis, phenotype-based filtering criteria, and confirmartory functional assays and Sanger sequencing, led to the identification of the underlying immune disorder (Figure 1). With this approach, eight of the overall group of variants resulted in potentially disease-causing mutations, distributed over seven patients.

We have divided NGS results into four categories: (I) genetic alterations associated with a canonical PID phenotype, (II) diagnostic genotype in atypical presentation, (III) genetic variants potentially involved in the immunological features, and (IV) multiple genetic variants, each of them was not *per se* causative individually of the disorder, even though the sum of variations of the different genes could be proven in the future of some pathogenic significance, as either causative or modifier factor.

The first and second groups included patients whose candidate genotypes were congruent with the immunologic features suggesting a causal relationship. Of note, exome sequencing allowed the identification of *PLDN* variants associated with a novel genetic cause of partial albinism and with PID (29). This study reported six patients who had undergone a diagnostic odyssey of 10–21 months driven by worldwide accepted protocols (40). Unfortunately, this candidate gene approach, although functionally driven, failed to yield a diagnosis. The diagnosis was finally made possible with NGS technology in a much more timely manner (2 months).

In the second category of patients, which included patients carrying genetic variants previously reported, but associated with novel features of previously established phenotypes, the molecular definition of the diagnosis contributed to expanding the overall knowledge of pathogenetic mechanisms underlying that specific disorder. In the patient with MYD88 deficiency, the atypical presentation was characterized by chronic yersiniosis resulting in terminal ileitis and recurrent neutropenia, in the absence of invasive pneumococcal disease, as is expected in this rare immunodeficiency. The atypical clinical presentation was responsible for the diagnostic delay. Moreover, Sanger sequencing of four candidate genes (*STAT3, ELANE, RAG1*, and *RAG2*) had given negative results (27). Homozygous mutations of *DOCK8* and *CLEC7A* were already reported as genetic cause of two distinct PIDs, such as autosomal recessive Hyper IgE syndrome and of CMC, respectively, but never observed in a single patient. It is likely that these genetic variants affecting two distinct loci, and probably concurring to the clinical manifestations of the patient, could not be identified without the availability of NGS (31).

The cases highlighted herein contribute to the explanation of several phenotypes for very rare disorders. Such broadening of the phenotypic spectrum is a phenomenon shared with many other rare disorders, as atypical patients are identified through NGS. In the case of congenital immune disorders, at the beginning only the most severe forms are described (41). Only after years from the initial description of the syndrome milder features of the disease are recognized or extra-immunological clinical signs identified.

In the third group, NGS sequencing revealed multiple heterozygous genetic variants in each subject, some of them potentially involved in the immunological features. All the variants of this group are either single heterozygous or *in cis*, thus further studied are needed, including WES or microarray testing to try to find the second mutation or uncover mutation(s) in a separate gene that fully explain(s) the phenotype. In the patient 012, the PRF1 gene alteration was proven to have functional relevance, since perforin expression in NK cells was reduced. However, the patient was not placed in the group 1 since the two heterozygous PRF1 variations were in *cis* at the Sanger sequencing. Previously, our group has documented that this heterozygous variation may act as a susceptibility cofactor, which under certain circumstances may be associated with a functional alteration (42).

Finally, the fourth group included patients showing multiple genotypic alterations associated with complex phenotypes that could not fit into defined clinical phenotypes. In this group, no genotype–phenotype relationship was possible. However, since these cases are extremely rare, these data should likewise be collected in a database. The creation of such a database might improve the interpretation of NGS results in those cases currently interpreted as no causative of the disorder. The identification of different individuals with the same phenotype and mutations in the same array of genes would suggest that the sum of variations in different genes exerts a pathogenic role, either as causative or as modifier factor.

However, even though in our study in 7 out of the 45 patients, a diagnosis was achieved, it should be considered that in the majority of the patients, target NGS approach did not allow to identify the genetic basis of the disease. Several aspects should be considered to interpret this observation. The first limitation is the limited number of the genes included on our targeted NGS panel. Moreover, the sequencing techniques do not provide enough coverage for intronic, promoter, or regulator regions. To overcome these technical limitations, whole exome or genome sequencing might be better strategies to deeply investigate these cases as the second-line diagnostic tool (14). Moreover, all NGS techniques, due to the generation of short reads, show a low sensitivity to detect complex structural variations (deletions, insertions, and inversions), repeat sequences, or complex rearrangement (43).

Based on these considerations, the identification of genetic defects in patients with PIDs is still a major challenge, and the functional implication of the variation must be considered mandatory to definitely prove the relationship between the genetic alteration and the related phenotype.

Despite the above mentioned limitations, NGS technology represents a cost-effective and rapid first-line genetic approach for the evaluation of complex cases of PIDs. The advantage of this technique is the simultaneous sequencing of a panel of genes, perhaps leading to the rapid identification of a diagnosis that may not have been otherwise considered using the traditional phenotype-driven approach. Overall, in spite of a moderately higher cost, at moment WES could represent the first-line approach to initial PID management. The sequential investigation of several candidate genes is, by comparison, a very time- and cost-consuming process. Prompt diagnosis shows an unquestioned clinical advantage, allowing initiation of appropriate and often life-saving treatment.

## Author Contributions

VG, GG, EC, VL, RC, and AP identified the pts and performed the immunological and phenotypic characterization; IT, EF, and CS performed the NGS and WES exps; RDA, AP, and VL performed the functional exps and the molecular confirmation exps; RB and CP interpreted the results; VG and CP wrote the manuscript; and RB and CP directed the project.

## Conflict of Interest Statement

The authors declare that the research was conducted in the absence of any commercial or financial relationships that could be construed as a potential conflict of interest. The reviewer JN and handling editor declared their shared affiliation, and the handling editor states that the process nevertheless met the standards of a fair and objective review.

## Footnotes

^1^http://evs.gs.washington.edu/EVS.

^2^http://exac.broadinstitute.org/.

## Abbreviations

ACMG, American College of Medical Genetics; ALPS, autoimmune lymphoproliferative syndrome; CMC, chronic mucocutaneous candidiasis; MYD88, myeloid differentiation factor 88; NGS, next-generation sequencing; PIDs, primary immunodeficiencies; SNVs, single nucleotide variants; TLR, toll-like receptor; T-NGS, targeted next-generation sequencing; WES, whole exome sequencing; WGS, whole genome sequencing.
